# Using Web-Based Content to Connect Young People With Real-life Mental Health Support: Qualitative Interview Study

**DOI:** 10.2196/38296

**Published:** 2023-01-04

**Authors:** Emily Adeane, Kerry Gibson

**Affiliations:** 1 School of Psychology The University of Auckland Auckland New Zealand

**Keywords:** young people, mental health, online help seeking, internet, mental health services, digital interviews

## Abstract

**Background:**

Young people experience high rates of mental health problems but make insufficient use of the formal services available to them. As young people are heavy users of the internet, there may be an untapped potential to use web-based content to encourage this hard-to-reach population to make better use of face-to-face mental health services. However, owing to the vast range of content available and the complexities in how young people engage with it, it is difficult to know what web-based content is most likely to resonate with this age group and facilitate their engagement with professional support.

**Objective:**

This study aimed to identify the types of web-based content young people identified as more likely to prompt youth engagement with mental health services.

**Methods:**

This study used a qualitative design conducted within a social constructionist epistemology that recognized the importance of youth empowerment in mental health. Digital interviews using WhatsApp instant messenger were conducted with 37 young people aged 16-23 years who participated as “expert informants” on the priorities and practices of youth in web-based spaces. The data were analyzed using reflexive thematic analysis to identify the types of web-based content that participants believed would encourage young people to reach out to a face-to-face mental health service for support.

**Results:**

The analysis generated 3 main themes related to the research question. First, participants noted that a lack of information about available services and how they worked prevented young people from engaging with face-to-face mental health services. They proposed web-based content that provided clear information about relevant mental health services and how to access them. They also suggested the use of both text and video to provide young people with greater insight into how face-to-face counseling might work. Second, participants recommended content dedicated to combating misconceptions about mental health and negative portrayals of mental health services and professionals that are prevalent in their web-based spaces. They suggested content that challenged the stigma surrounding mental health and help seeking and highlighted the value of mental health services. Finally, participants suggested that young people would be more likely to respond to “relatable” digital stories of using mental health services, recounted in the context of a personal connection with someone they trusted.

**Conclusions:**

This study offers recommendations for professionals and service providers on how to better engage young people with real-life mental health support using web-based content. Web-based content can be used to challenge some of the barriers that continue to prevent young people from accessing face-to-face mental health services and underlines the importance of including young people’s voices in the design of web-based mental health content.

## Introduction

Young people experience high rates of mental health problems; however, <20% of those in need will access professional help [1,2]. In New Zealand and other similar countries, there is a range of services available to help young people (defined by the United Nations as being aged between 15 and 24 years) [3] with mental health problems, including those offered by health care providers, schools or universities, and community-based organizations. However, researchers have recognized that there are a number of practical, psychological, and social barriers that prevent young people from reaching out to these supports, including a lack of knowledge about what services are available, prior negative experiences of help seeking, concerns about the stigma attached to having a mental health problem, and a preference for self-reliance in dealing with their difficulties [4,5]. Due to these barriers, many young people do not seek help when they need it, including (and especially) individuals experiencing suicidality and those from marginalized communities [6]. Early intervention is key to preventing long-term mental health problems among young people, and there is a need to explore creative strategies to overcome the barriers that prevent young people from getting the help they need [7,8].

Given young people’s high internet use, there is a growing awareness of the potential for this platform to provide mental health information and resources that might facilitate this hard-to-reach group’s access to mental health support [9]. Despite their reluctance to seek help for mental health problems in *real life*, many young people are willing to look on the web, with surveys suggesting that 30% to 50% of young people have used the internet to access psychological information or support [10-12]. As professionals recognize the potential of web-based resources to reach young people in distress, there is a burgeoning number of internet interventions available to youth populations. Young people are now able to receive professional support on the web, including through video, text, or email counseling [13,14], or via tools such as moderated peer forums [15]. Youth may also access cognitive behavioral therapy courses through apps [16] or games such as *SPARX*, created by researchers at the University of Auckland [17].

These web-based mental health supports have been welcomed for their ability to reduce clinician burden and offer relatively equal treatment access to individuals, regardless of location, mobility, or economic status [18]. However, it has also been recognized that web-based interventions might not be suitable for all young people. For some young people, web-based support might be inappropriate because of their complex profiles of risk and regular disengagement [19]; for other young people, face-to-face counseling might be a preference [20]. Recent research has called for increased recognition of the role that web-based resources can play in helping young people access the full range of mental health support available to them, both in the virtual and real world [9].

Although most research in recent years has focused on the design and evaluation of internet-based interventions, less attention has been paid to the potential of the internet to facilitate young people’s access to face-to-face mental health support. However, some existing research indicates that web-based education and support can serve as a valuable conduit to help young people connect to formal mental health services in their local areas [21-24]. For example, studies have shown that web-based tools can help young people identify when they have a mental health problem [25], and informational websites can provide encouragement and guidance on seeking professional help [26-28].

However, young people’s web-based engagement with mental health information extends well beyond these professional informational resources, with many young people choosing to use informal social media networks instead [29]. Although there is some evidence of peers encouraging harmful practices related to mental health [30], these informal networks might also provide peer encouragement to seek professional help [31]. These studies suggest that both formal and informal web-based engagements have the potential to facilitate offline mental health help seeking, at least some of the time.

Ito et al [32] argued that young people do not use different parts of the internet in isolation. Instead, similar to patrons at a buffet, they take content from various stations, which may vary in terms of privacy, modality, or formality [32]. The mental health information available to young people ranges from professionally curated material on static websites to peer-produced videos on YouTube or TikTok and more personal and private Facebook or Instagram messenger groups. A recent review of young people’s preferences for mental health information suggested that they often relied on search engines to explore a wide range of resources available, that they tended to use formal professional websites less often than other sources, and that they also made use of a variety of informal discussion forums and other web-based communities to gain information [9]. In terms of specific site features that are valued by young people, interactive functionality and social networking capabilities appear to be key [33]. Youth also engage with mental health content more if it is multimedia or through YouTube and contains personal experiences, humor, or detailed information about what treatment looks like [34]. However, there is still much to be learnt about the types of content that are most likely to resonate with this age group and facilitate their engagement with *real-life* support.

It is important to understand which resources young people attend to in their web-based world, what ideas and modes of presentation this generation of youth are more likely to resonate with, and what specific content would encourage them to seek professional mental health support. To do this, it is vital to understand young people’s own web-based practices and preferences for mental health content.

It is common for research to investigate young people’s engagement with a particular mental health website or tool to determine its impact [35]. However, given the wide array of web-based sources and modalities available to young people seeking support or information on mental health, exploring the impact of a particular set of web-based resources may miss the complex and fluid way young people engage in this terrain. Another obstacle to understanding young people’s preferences for mental health content is that adults, including professionals, are often deliberately excluded from young people’s virtual worlds. Young people value these web-based spaces precisely because they provide protection and privacy from adult intrusion [32]. In the largely hidden worlds of young people’s internet use, it is difficult to know what types of content they are attending to and which they ignore. The only way to mediate this lack of access to young people’s web-based worlds is to engage their own expertise and determine what types of content they connect to on the web and what they take from these engagements.

Qualitative methodologies provide a way of accessing young people’s own accounts of their experience, with the advantage of placing them in a meaningful context [36]. However, there are several hurdles to overcome when researching young people’s preferences and practices in the web-based environment. Power imbalances plague youth research and often prevent young people from engaging openly with researchers [37]. Furthermore, different data collection methods with young people pose unique challenges. Face-to-face interviews, in particular, present problems of access for young people who might have limited independence, mobility, and time [38]. It can also be difficult for young people to accurately convey the reality of web-based practices through verbal interviews conducted face-to-face or on the phone. In addition, in the sensitive area of mental health research, the same barriers that prevent young people from reaching out to professional support may influence the extent to which they will speak openly about these things to researchers [39]. Using familiar digital technologies to interview young people may help them feel more comfortable sharing information about their web-based practices, especially in relation to mental health [40].

Existing literature in this area suggests that the internet may offer the potential to facilitate young people’s engagement with mental health support. However, it also demonstrates that we need to understand more about the types of mental health resources young people encounter in their complex web-based lives, which resources they find compelling, and which are more likely to facilitate their willingness to reach out for mental health support *in real life*. To maximize the potential of the internet to facilitate young people’s access to help, it is vital to match mental health messaging to this generation’s priorities and concerns [41].

## Methods

### Research Design

This research draws on young people’s knowledge of youth mental health practices to address the following research question: “What types of web-based content do young people identify as being more likely to encourage them and their peers to use ‘in real-life’ mental health services?”

The study was designed to explore the web-based worlds of young people, using youth informants to provide insights into their own preferences and practices, and those they observed among other young people in their web-based networks. The study used a novel digital interview method using WhatsApp instant messaging to facilitate young people’s comfort in sharing information on the sensitive topic of mental health and their ease of describing web-based content and practices.

This research is underpinned by a social constructionist epistemology that recognizes that the terrain of mental health might be interpreted within youth culture in ways that are different from professional views [29,41]. It also draws from a youth empowerment approach that aims to amplify young people’s voices on key issues that affect them [42]. The researchers used reflexive thematic analysis, a theoretically flexible model that is consistent with these underlying paradigms and that allows researchers to explore commonalities in people’s experiences across a number of participants [43]. This approach asserts the importance of recognizing the impact of a researcher’s identity and positioning with regard to the research itself. The first author is studying to be a clinical psychologist but also related to participants as a young person and fellow “digital native” [44]. The second author is a clinical psychologist and researcher who approached the topic from outside the participants’ age demographic. As a research team, we were conscious of the need to challenge our own professional and adult assumptions and make space for the views of the young people participating in our study.

### Recruitment

We recruited participants through youth development agencies, youth groups, and youth social media networks, for example, Instagram and Facebook pages belonging to individuals and organizations. Potential participants needed to be aged between 16 and 23 years, based in New Zealand, and identify as regular internet users. We wanted participants who could serve as “expert informants” on young people’s digital practices regarding mental health. As our focus was eliciting participants’ observations about practices in their web-based networks, we did not require that they have a direct experience of mental health problems themselves. Instead, we asked for participants who had “an interest in youth mental health.” However, it was clear from the interviews that many of those who participated in the study had used web-based resources to help them with their own mental health. Young people who were interested to participate in the study contacted the researchers through email or a dedicated research phone. Potential participants were then provided with the necessary information and the opportunity to ask questions. Immediately before beginning the interview, researchers messaged each participant on the WhatsApp platform to confirm whether they had read the information and consent forms, had any questions, and agreed to participate. An affirmative written response from each participant (eg, “yes I consent”) was included in each transcript as a proof of informed consent. The researchers then collected demographic information (age, gender, sexuality, ethnicity, occupation, and location) and proceeded with the interview. Participants were offered a NZ $30 (US $19) gift voucher as compensation.

### Participants

A total of 37 young adults participated in this study. Participants were aged 16-23 years, with all but 6 participants aged <20 years. Of these 37 participants, 24 (65%) identified as women, 12 (32%) as men, and 1 (3%) as nonbinary. There were 67% (25/37) heterosexual, 11% (4/37) bisexual, 8% (3/37) gay, and 3% (1/37) asexual participants. A further 11% (4/37) participants chose not to disclose their sexuality. The participants self-described their ethnicity, resulting in overlapping data for several participants and no data for 1 individual. Of the 37 participants, 21 (57%) were identified as New Zealand European, 5 (13%) as Māori, 5 (13%) as Asian, and 2 (5%) as Pasifika. Furthermore, 3 (8%) participants were from the Middle East, 3 (8%) from Africa, and 2 (5%) from other European countries. Most of the participants resided in large cities in New Zealand, with only 5% (2/37) participants from smaller towns. The researchers judged the overall body of data to be sufficient for the aim and type of this research [45].

### Data Collection

We conducted interviews using the instant messaging platform WhatsApp. Our previous use of this data collection methodology was found to facilitate young people’s comfort in talking about the sensitive issue of mental health and allowed participants to accurately represent their web-based practices through digital communication such as textspeak, emojis, and screenshots [46]. Reflections on the experience of using WhatsApp as a medium to conduct these interviews have been described elsewhere [40,46]. Interviews took place between September 2019 and May 2020 and lasted between 45 and 105 minutes.

The interviews followed a semistructured guide, with open questions designed to elicit responses about young people’s engagement with mental health resources in their web-based environments. We asked participants to reflect on their own experience as well as the practices of others in their web-based networks, using questions such as “What mental health resources do you use?” “What makes young people attend to these resources?” “What stops young people from using mental health services irl [in real life]?” and “What content would make you or others more likely to use these services?” We also used a range of prompts, including requests for examples or further explanation, and encouraged participants to share screenshots of relevant web-based content if desired.

### Data Analysis

Researchers exported both written and nontextual interview data into transcripts, removed identifying information, and then stored them on a secure university computer. These transcripts were analyzed using reflexive thematic analysis, which recommends a recursive method for identifying themes or patterns of meaning [43,47]. Transcripts were read repeatedly for data familiarization and were examined in relation to the central research question. We labeled relevant statements with short descriptive codes such as “relatable” or “easy to find.” These initial ideas were collated, and their connections were visually mapped out. We identified tentative themes that were then refined recursively and collaboratively within the research team. Analytic decisions were reached via consensus, a quality assurance process consistent with the study’s underlying epistemology [48,49]. The language used by participants in their digital communication was reproduced in its unedited form to maintain the authenticity of the data. Nontextual data such as screenshots, emojis, and pictures were considered within the context of the written interview data and, where appropriate, were coded and labeled in a similar fashion. This content was reported in the results through the use of image descriptions or reproductions of written text present in screenshots (eg, a comment under a YouTube video).

### Ethics Approval

Mental health is a sensitive issue, and ethical considerations were at the forefront of our minds in planning and conducting this research. Information sheets provided to the participants before the interviews outlined the potential risks of participation and information on support services. The interviews were conducted under the supervision of the second author (KG) who is a clinical psychologist with experience in youth mental health. Interviewers monitored the participants’ responses for signs of distress and were prepared to reprovide information on support services as needed. The University of Auckland Human Participants Ethics Committee approved this study (reference 023698).

There were some ethical challenges raised by the use of the WhatsApp interview method. The researchers clarified with the participants that the WhatsApp interview would take place at a particular time, but they were able to add comments for 14 days afterward. They were also encouraged to review their transcript to check whether they wanted to edit or remove any information. If participants chose to include a screenshot from their social media networks to illustrate a point, it was edited to remove all identifying information and stored with the transcript.

## Results

### Overview

A total of 3 themes and 7 subthemes were identified in the data. The 3 main themes each refer to the different types of content that participants identified as being likely to facilitate young people’s engagement with face-to-face mental health services: first, content that provided clear information about mental health services; second, content that challenged misconceptions surrounding the use of mental health services; and finally, content that provided a relatable experience with someone who had used mental health services.

### Information About Mental Health Services

This theme captured participants’ strong desire for improvements to the quality, scope, and presentation of web-based information regarding how to access face-to-face mental health services and how counseling actually works.

#### How to Access Counseling: “A Step-by-Step Guide”

This subtheme highlighted young people’s lack of knowledge about mental health services and the barrier this posed to accessing professional support. Participants explained that psychoeducational websites often simply advised young people to just “speak to a doctor or counsellor” [P17] with the assumption that they would know how to go about doing this. Rather than a simple task, participants pointed out that young people actually had to gather a large amount of information for each potential service before even beginning to connect with them, including the basic details such as who; what; where; as well as costs involved, availability, and admission criteria. One participant captured how daunting this fact-finding mission could be, leaving them feeling like “i literally wouldn’t know where to start with finding a therapist” [P21].

Participants also highlighted the difficulties of knowing how to sift through all of the web-based information about mental health services once they had begun searching for it. They acknowledged that a vast amount of information could be found on the web but noted that this created difficulties for them in filtering out what was relevant or appropriate to their situation. Rather than facilitating help seeking, the deluge of information seemed to instead put young people off engaging with mental health services, as one participant explained:

It’s interesting because in truth there is already a lot of info online. If I were to make the right searches I’d be bombarded with all of this info which would supposedly help me...however, I feel as though it’s more about the way the info is packaged. For first time info getters, it may seem a bit overwhelming, and prevent us from going back.

P13

Participants suggested that rather than being bombarded with information, they and other young people would prefer information that directed them to relevant resources for their age and situation:

[Its] just that so many options come up and I don’t know who is good and who is not and for someone my age with not much money the free options tend to not show up very much. If there was a resource that could show me people to go to I would be far more likely to actually contact someone and get help before I get to a low point where I’m desperate for help again.

P34

Participants’ preferences for targeted resources also extended to geographical considerations. They noted that a particular disadvantage of web-based material is that it does not necessarily take geographical boundaries into account, and they wanted to know which services were available in their local community. As one participant elaborated:

they’re a bit hard to learn about those ones online because every community has different resources available to you...[it would help] if it was clear what support was available to the young people in your community.

P11

Perhaps reflecting previous failed attempts at help seeking, some participants emphasized that they also wanted reassurance that a particular service would be willing to admit them and that they would not find themselves facing additional barriers such as not meeting admission criteria or unaffordable fees.

Participants also explained that they wanted content that provided “quick answers” to their questions about mental health support and preferred sites that provided up-front information or gave real-time responses to queries. One participant captured this in their comment that they wanted “a service that gave me fast results, for example an online chat or a FAQ, rather than an email that could take days to be responded to” [P20].

Furthermore, young people not only wanted information that would lead them to appropriate mental health services but they also wanted detailed guidance on how to access these, for example, “a clear pathway to get access to that specific support. So even if you know it’s there, you know how to use it” [P11]. Participants noted that the practical and administrative tasks associated with seeking professional support could be very daunting for a young person in distress and suggested “probably the biggest help would be like a step-by-step guide for who to call, how to pay, etc.” [P21].

The task of booking therapy appointments was seen as a hurdle for many participants, especially when this involved speaking over the phone as one young person explained:

a huge, huge aspect of the process that I think turns people off is having to call to book.

P37

Another participant suggested how web-based resources could make the booking experience less intimidating for young people and therefore increase their chances of actually reaching out, saying if “booking an appt was easy and efficient (ideally online through a form), that would be good in encouraging that young person to go ahead with it” [P31].

#### How Counseling Works: “Knowing What it Is Actually Like”

This subtheme highlighted young people’s need for more information about what would be required of them if they attended a face-to-face mental health service. Formal mental health support was depicted as an unfamiliar terrain for most young people, with many relying on the little they had gleaned from popular media to inform their perspectives as one participant explained:

My mind can’t help but think of scenes from movies and TV for examples since I know so little about counselling in general.

P23

Participants said that more detailed information about how counseling worked could help to challenge some of the misconceptions and the mysteries surrounding mental health and counseling in youth networks:

if there are resources online that describe the process of visiting counselling services, I think it would take some of the fear or stigma away that would otherwise prevent younger people from seeing it as a viable option for them.

P7

Participants suggested that web-based information could, for example, help demystify what happened in the first session of counseling or tell young people more about how the process of counseling worked in general. Speaking openly with a professional about distress was particularly intimidating. One participant recommended that web-based videos of simulated counseling conversations might help young people see how counselors guide the conversation so “you don’t have to worry that you won’t have anything to talk about” [P37].

In addition to not knowing what to say, participants noted that young people were unsure about how they were expected to behave when they attended a mental health service. One participant explained that because counseling was so unlike their other experiences, they were anxious about what would be required of them in this setting:

I think I would only use it if I was...informed on how to act or something? For me it’s a very non ordinary experience so I’m not sure what the expected “etiquette” or something is. Basically knowing what face-to-face counselling is actually like.

P23

Participants also noted the potential for initial web-based contact with a counseling service to give young people a reassuring taste of what it might be like to attend in person, with one saying:

I think having online services would be a great idea so that people can have counselling online until they are comfortable with the idea of going in person, as I know for some people that scares them.

P22

### Challenging Misconceptions About Mental Health Services

This second theme identified participants’ ideas for using web-based content to combat some of the stigma and misconceptions surrounding mental health help seeking that could prevent young people from reaching out for mental health support.

#### Encourage Recognition of the Need for Support: “They Are Worthwhile and Deserve Help”

This subtheme captured the recommendation for content that could challenge young people’s perceptions that they were not entitled to mental health support. The reluctance of young people to use mental health services was partly ascribed to a concern that their problems were not valid or sufficiently serious to warrant professional help. One participant illustrated this with a screenshot of a YouTube comment that stated:

young people have been raised to believe their problems don’t matter and aren’t worth sharing with people.

P14

Another participant explained that young people felt a need to evaluate whether their problem was “big enough” to deserve support and that they might “think that their problems are minor and normal and that by asking for help they would be being dramatic” [P22].

Participants also explained that some young people might feel like they were burdensome or unworthy of support. As one participant explained, young people are “not wanting to trouble anyone. A lot of people in that headspace don’t feel worthy of anybody else’s time” [P5]. Against the context of these obstacles to young people seeking help, participants explained that it was important that web-based resources give young people the explicit message “that they’re not a burden and they are worthy of help...just letting them know that they are a priority, and their wellbeing is important” [P36].

#### Destigmatize Professional Help: “Normalizing Going to Counseling”

Participants conveyed the importance of addressing the stigma that they felt still surrounded mental health. Almost all participants mentioned stigma as a key barrier to professional support seeking and felt that web-based content needed to address this obstacle to facilitate young people’s engagement with mental health services. One participant suggested using statistics to challenge the idea that help seeking was unusual, explaining:

I think knowing how normal it is for people to seek offline support would help! It’s always a hard step to take but once you realise how common it is for people to seek offline help it makes it easier.

P4

Other participants suggested that if more young people were seen “speaking about [their] experiences online where it can reach a lot of people,” that this may help with “normalising going to counselling or seeing a therapist” [P7]. Another participant explained how content with an undramatic and more normalizing tone may also be less likely to unnerve young people. As one participant explained:

I believe that if the info just began in a very calm way and discussed how we could potentially reach out in real life/offline, young people would feel like getting help offline can be achieved.

P13

Participants explained that to normalize help seeking, approaching a mental health service should be treated as an “ordinary,” everyday task. One participant’s suggestion reiterated this idea for “something that doesn’t make it out as a big deal” [P20]. Another participant suggested that it might be possible to reduce the “seriousness” of web-based mental health information by using humorous messaging. As she explained, mental health information may be more engaging for young people “if it was put into meme form, I think young people genuinely find it easier to connect with difficult thing[s] through humour” [P36].

Participants also suggested that mental health services use images or short-form video-based platforms such as Instagram and TikTok so that content about mental health services would seem more familiar and less alienating. One participant explained that this was because such pages were “colourful and look like any other Instagram story post so you don’t feel like you’re having to go to a counsellor and they don’t make you anxious” [P18].

#### Promote the Value of Mental Health Services: “The Importance of Face-to-face”

This subtheme conveyed the recommendation that web-based content should be used to counter negative views of mental health services and promote more positive representations. Some of the participants described encountering off-putting descriptions of mental health professionals and counseling in their web-based environments. For example, one participant provided this account:

Like, I’d be scrolling through Twitter and see a post of how bad psychiatrist are and they just want your money and to have a laugh. You need to fix yourself and psychiatrists are fake.

P17

Participants suggested that web-based content that actively challenges some of these negative perceptions and accounts would be valuable to convince young people that accessing mental health support is a safe and worthwhile activity.

Participants also explained how web-based descriptions of negative experiences with therapy could make young people question the efficacy of mental health services and dissuade them from trying it for themselves. As one participant explained, a barrier for her seeking help was hearing about “how it doesn’t work for some people or others would have something bad to say about it which would make me worry about wanting to do it as well” [P35]. A simple solution to this was suggested by another participant, who advocated for more “positive stories from people who have been to see a counsellor” [P18], which they felt would make young people feel hopeful that therapy could work for them too.

Participants also suggested that young people accustomed to finding mental health information and informal support on the web might be less convinced that there was any further value in using mental health services in the real world. As one interviewee put it, “they may not understand that its possibly very important for them to have face to face help” [P1]. Participants felt that it could therefore be helpful to promote the value of these services, for example:

information about the importance of face-to-face meetings and how it positively effects the mental health e.g. promotions.

P14

This participant went on to suggest that this could be backed up by “research or study showing that it helps, like statistics etc” that might convince young people that it was worth trying. Participants also suggested that these types of content might need to actively challenge young people’s reluctance to leave the safety and familiarity of their web-based support seeking. One participant elaborated, for example, that “listing the benefits of the offline support as opposed to online” would be a helpful strategy [P6].

### Relatable Experience With Mental Health Services

This theme captures participants’ preferences for receiving web-based mental health content from people they can relate to and trust. This includes positive accounts provided by other young people who have used counseling as well as influencers and celebrities.

#### Accounts of Young People’s Positive Experiences of Counseling: “Allow Young People to Relate to Them”

This subtheme describes the importance that participants ascribed to hearing positive stories from other young people who had used face-to-face mental health services.

Rather than responding to slick advertising, participants spoke about how they were more inclined to respond to content if it seemed “relatable” or came from a person they knew. They said that young people would be more likely to approach face-to-face services if they could “hear experiences or personal stories of the topic they need aid in” [P24]. They felt that this would help people feel less isolated, as explained by one participant who referred to a community mental health service with a strong web-based presence which “has lots of stories of other people’s experience so you know you’re not alone” [P11].

Participants described how this relatable content made it easier for young people to imagine themselves getting help and, therefore, may increase their chances of seeking it. As one participant suggested:

Maybe just making posts that shared different people’s stories or perspectives related to mental health which would allow young people to relate to them and realise there is support out there.

P9

In addition to general therapy stories, participants wanted to hear from individuals who had actually attended the particular service they were considering. One participant explained that hearing from someone relatable would help establish the service as somewhere trustworthy:

I think once again being able to hear about people’s experiences with them would be a great way to persuade people. It’s hard to try something new or talk to someone new when you don’t know whether that specific person or their company has worked for others before.

P35

Participants also wanted the ability to interact and ask questions through “talking to other people who’ve used the same service” [P6] through web-based messaging systems. Some suggested that this might be done anonymously to protect the identity of people who had used mental health services, whereas others suggested asking “friends who i know go to therapy what they did” [P21].

Participants noted that young people were often extremely skeptical of content from unfamiliar people or organizations and were likely to respond better to content from people they knew and trusted. They noted that youth were suspicious of any web-based material that appeared to reflect corporate or commercial interests and experienced digital marketing as annoying and invasive. As one participant put it:

When you’re in your downtime on Facebook or YouTube. Regardless of how important or good the advertised service is, nobody likes ads.

P31

Participants emphasized that young people needed to trust the person or organization delivering the message if they were to respond to it. One participant explained:

Probably the biggest thing would be friends supporting me in using it...Most of my contact with friends be them people I know in real life, or online ones, is through private messages and conversations. So someone I trust in this way telling me they support me seeking out further help, or maybe even offering it as a potential solution would have the biggest chance of me probably using it.

P2

#### Endorsements From Influencers and Celebrities: “People They Feel Like They Look up to but Also Relate to”

In their interviews, participants often suggested that celebrities, other individuals with large web-based followings, and even popular “pages” could have a powerful impact on whether young people seek mental health support. For example, it was proposed that if these “influencers” openly acknowledged that they had been to counseling, their young followers might see it as a more acceptable thing to do. Although help-seeking stories from peers could be helpful, it was felt that this content would carry even more weight coming from influencers, who combine the relatability of peers with the prestige of celebrities. As one participant explained:

for young people I’d say they’d need celebrities or youtubers, people they feel like they look up to but also relate to.

P7

Popular users of the video-streaming site YouTube (“YouTubers”) were seen as particularly easy to identify with “because they’re people similar to oneself talking about their relatable experiences so they also sound like a friend” [P18]. The use of videos appears essential in allowing young people to trust and relate to YouTubers. One young person captured the sense of intimacy and authenticity conferred by this medium, explaining that this was “because videos on YouTube makes the person feel he/she is sitting with the other one. They see and hear them. And plus they know that person is a real person” [P14].

However, some individuals expressed concerns about popular accounts giving these sorts of endorsements, suggesting that some influencers may have ulterior motives:

I think to be careful who you take advice from, there’re lots of people online who say if you buy their product/plan/whatever it’ll fix all your problems and sometimes it’s hard to know who you can trust.

P21

Participants also felt that young people would be wary of recommendations coming from newly created accounts and that services must instead go through influencers who have already built up a trusting relationship with their followers. As one participant put it:

Yeah probably, it’d help if they were shared by established people/groups. Like, people can go and see that its not the only thing they’ve posted and they’re not just trying to sell something.

P36

## Discussion

### Principal Findings

There is an underrealized potential for the internet to support young people to use the face-to-face services that some might need. Although resources on the internet have the potential to facilitate young people’s access to mental health services in the real world, to be effective, these need to connect with the priorities and concerns of contemporary youth. This study provides important insights into the types of content that might be more likely to prompt young people to use mental health resources *in real life*.

Low awareness of services has previously been recognized as a barrier to help seeking [50]. However, given that young people have access to a vast array of resources on the internet, it is perhaps surprising that they still felt that a lack of basic information (such as the types and availability of local services, eligibility criteria, and contact details) was an obstacle for them to reach out for help. This anomaly seems to be a function of more general challenges associated with sifting through and evaluating the vast amount of information available on the web [51]. Web-based directories or tools that help young people identify personally relevant services may go some way to addressing this obstacle to help seeking. However, young people’s struggle to find accessible mental health services in their local areas may also reflect the ongoing practical barriers that put some of these services out of the reach of many young people in New Zealand [52].

Furthermore, despite the broad public awareness of counseling and other related mental health services in Western countries such as New Zealand, young people might still struggle to envisage precisely how these things work [50,53,54]. A consequence of incomplete knowledge about what actually happens in a mental health service is that young people may be more susceptible to harmful myths or inaccurate fictional representations of mental health care that form part of their media world [55,56]. However, the same digital technology that produces these myths may also offer unique opportunities to provide young people with more accurate representations of how a mental health service works. For example, videos introducing staff members and touring the physical environment of a service, or re-enacting different components of the engagement, including counseling sessions, might help break down the barriers that prevent young people from using these services. Young people’s comfort and familiarity with web-based interactions may also provide a valuable opportunity to allow them to “try out” aspects of a mental health service before they find the confidence to use it. Other researchers have acknowledged the value of web-based services as a way for young people to familiarize themselves with the experience of counseling before making the leap to *real-life* mental health services [26,27]. The opportunity to actually see and interact with the same mental health professionals that they might later see offline would likely enhance this effect.

The findings of this study also reflect the priority that this generation gives to the immediacy of access to information and support, leading some to describe them as having “fast-twitch wiring” [57]. For young people, mental health information and support must be available instantly and in the moment of need [41]. It would be valuable for mental health services to use the internet to help young people quickly and easily book an appointment and transition to offline services, if needed.

Our study also suggests that the internet could be used to challenge some of the negative representations of mental health and help seeking that prevent young people from reaching out to mental health services for support. It is important to counteract young people’s perceptions that their problems are not “big enough” for support or that they would burden others by doing this. This recommendation aligns with campaigns that tell young people that they do not have to put up with distress and that no problem is undeserving of support [50,58]. Stigma is a well-established barrier to mental health help seeking, and unsurprisingly, the participants in this study were clearly aware of its impact on young people [39,50,58]. Interestingly, participants’ suggestions for addressing stigma challenge the conventional illness representations of mental health commonly used in public health promotion, such as “mental illness is an illness like any other” [59]. Instead, participants emphasized that experiencing distress and using mental health services should not be pathologized but rather treated as a normal and ordinary experience for young people. Of particular interest were suggestions that using the short-form video and image-based formats ubiquitous on youth social media networks could help normalize mental health service use. This is consistent with the widely held view that health messaging is more effective when it resonates with people’s everyday practices and common ways of communicating [60]. The suggestion that humorous memes could be used to dilute the “seriousness” of mental health messaging may raise additional challenges. In a study by Robinson et al [61], young people rated suicide prevention materials with a humorous tone as the least acceptable and having the lowest chance of persuading them to seek support. In contrast, Martini et al [34] found that content from Buzzfeed’s mental health week had higher engagement when humor was used. It would be important to find the right way to present mental health issues using humor, and more research might be needed to establish exactly how this could work.

For the young people in this study, web-based resources were seen as offering an opportunity to promote the value of face-to-face mental health support. Young people often underestimate the need for support and may not recognize its potential value to them [2]. However, there are likely to be unique challenges in engaging a generation that might need to be persuaded that there are benefits to real-world interactions that cannot be provided on the web [33]. Having information available on the web that outlines the benefits of using face-to-face mental health services may go some way toward remedying this [58,62]. Our research also highlights the importance of directly comparing the benefits of offline mental health services with the support and information young people can access on the web. Young people often cite that the “distance” and feeling of (apparent or actual) anonymity make web-based mental health discussions easier [13]. They may need strong encouragement to leave the comfort, familiarity, and apparent safety of a web-based environment and confront the challenges of face-to-face discussions about their mental health.

One of the most significant findings of this study was the importance of delivering content to young people in forms that were “relatable.” Young people in this generation are highly aware of the importance of authenticity and tuned into the potential for content and people who are “fake” [41]. Unsurprisingly, young people’s help-seeking behavior was strongly influenced by concerns about trust. Research conducted by the authors of this study has emphasized that young people are only willing to engage with mental health content on the web if they feel they can trust the person they are communicating with [46]. Young people have been noted to be more inclined to turn to their peers for advice on mental health issues, whether this be offline or on the web [41]. Consistent with existing research, this study suggests the value of using digital stories from peers who are seen as relatable and trustworthy to communicate the value of reaching out for mental health support. Many young people in this study saw influencers as trustworthy and relatable, as has also been shown in other research [63,64]. YouTubers were seen as particularly authentic owing to the “illusions of intimacy” they create through the use of video [64]. Although celebrity endorsements are not new, social media influencers appear to have a unique appeal, combining the relatability of peers and the perceived expertise and prestige of celebrities [65]. The use of web-based influencers to promote face-to-face mental health services is a developing area that holds considerable promise for promoting youth mental health [66-69]. Professionals should carefully consider how they might enlist local influencers to help them promote their mental health services and use their trusting relationships with young people to encourage their use.

In terms of methodology, this study demonstrates that instant messaging interviews are an accessible and effective way to gain an understanding of young people’s web-based practices [40].

### Limitations

Although this research captured some of the diversity in New Zealand’s young people, there was not a high number of Māori and Pasifika participants [70]. This is a particular limitation given that this group may be less inclined to use mental health services because of the history of colonization and exposure to systemic racism [71]. This may limit the findings of this particular analysis, especially considering that these cultural groups appear to have different help-seeking behaviors [72]. It is also worth noting that this study primarily reflects the experiences and preferences of urban young people, which may be different from those living in rural areas [73]. Consistent with the tenants of reflexivity in thematic analysis, we acknowledge how our professional and personal backgrounds as researchers will have affected and enriched the entire research process, from conception to analysis. For example, this may be seen in our tendency toward eliciting and highlighting participants’ perspectives, as they relate to mental ill health and distress rather than well-being, which likely stems from our experiences within clinical psychology.

### Conclusions

This study contributes to the ongoing efforts to make effective use of the internet to support young people’s mental health. It provides important insights into the types of web-based content that young people believe would facilitate their and their peers’ engagement with face-to-face mental health services. The findings indicate that to connect with young people, web-based mental health content should provide relevant and detailed information about services and how to use them. Web-based content might also actively counter some of the psychological and social barriers that prevent young people from using mental health services. One of the most important messages for service providers or others wanting to reach young people in distress is acknowledging the unique importance of trust in engaging with this group and mobilizing influencers and peer networks to facilitate greater access to help. The internet has rich resources for promoting mental health among young people. However, professionals need to learn from young people about what matters to them to make the most of this opportunity to engage young people with professional support when they need it.
